# Comparison of Characteristics of Deaths From Drug Overdose Before vs During the COVID-19 Pandemic in Rhode Island

**DOI:** 10.1001/jamanetworkopen.2021.25538

**Published:** 2021-09-17

**Authors:** Alexandria Macmadu, Sivakumar Batthala, Annice M. Correia Gabel, Marti Rosenberg, Rik Ganguly, Jesse L. Yedinak, Benjamin D. Hallowell, Rachel P. Scagos, Elizabeth A. Samuels, Magdalena Cerdá, Kimberly Paull, Brandon D. L. Marshall

**Affiliations:** 1 Department of Epidemiology, Brown University School of Public Health, Providence, Rhode Island; 2 Executive Office of Health and Human Services, State of Rhode Island, Cranston; 3 Center for Health Data and Analysis, Rhode Island Department of Health, Providence; 4 Department of Emergency Medicine, Alpert Medical School of Brown University, Providence, Rhode Island; 5 Division of Epidemiology, Department of Population Health, Center for Opioid Epidemiology and Policy, School of Medicine, New York University, New York

## Abstract

**Importance:**

The rate of deaths from overdose has increased during the COVID-19 pandemic, and recent US overdose mortality rates have been markedly high. However, scant data are available on the causes of this increase or subpopulations at elevated risk.

**Objective:**

To evaluate the rates and characteristics of deaths from drug overdose before vs during the COVID-19 pandemic.

**Design, Setting, and Participants:**

This retrospective, population-based cohort study used data from 4 statewide databases linked at the person level via the Rhode Island Data Ecosystem on adults with deaths due to overdose in Rhode Island from January 1 to August 31, 2019, and January 1 to August 31, 2020.

**Main Outcomes and Measures:**

The rates of unintentional deaths from drug-related overdose during the 2019 and 2020 observation periods overall and by sociodemographic characteristics, drugs contributing to the cause of death, location of death, and socioeconomic factors were evaluated. In subgroup analyses restricted to Medicaid beneficiaries (n = 271), the proportions of deaths from overdose by behavioral health treatment and diagnosis claims in the year before death were also examined.

**Results:**

A total of 470 adults who died of drug overdose were included in the analysis (353 men [75%]; mean [SD] age, 43.5 [12.1] years). The rate of deaths from overdose in Rhode Island increased 28.1%, from 29.2 per 100 000 person-years in 2019 to 37.4 per 100 000 person-years in 2020 (*P* = .009). Compared with 2019, rates of deaths due to overdose during 2020 were higher among men (43.2 vs 59.2 per 100 000 person-years; *P* = .003), non-Hispanic White individuals (31.0 vs 42.0 per 100 000 person-years; *P* = .005), single individuals (54.8 vs 70.4 per 100 000 person-years; *P* = .04), deaths involving synthetic opioids (20.8 vs 28.3 per 100 000 person-years; *P* = .005), and deaths occurring in a personal residence (13.2 vs 19.7 per 100 000 person-years; *P* = .003). A decrease in the proportion of deaths from overdose involving heroin (11 of 206 [5%] vs <2% [exact value suppressed]; *P* = .02) and an increase among persons experiencing job loss (16 of 206 [8%] vs 41 of 264 [16%]; *P* = .01) from 2019 to 2020 were observed. Among individuals who died of overdose and were Medicaid beneficiaries, the proportions of those aged 50 to 59 years with anxiety (11 of 121 [9%] vs 29 of 150 [19%]; *P* = .03), men with depression (27 of 121 [22%] vs 57 of 150 [38%]; *P* = .008), and men with anxiety (28 of 121 [23%] vs 55 of 150 [37%]; *P* = .02) increased during 2020 compared with 2019.

**Conclusions and Relevance:**

In this cohort study, during the first 8 months of 2020, the rate of deaths from overdose increased in Rhode Island compared with the same period in 2019, and several emerging characteristics of deaths from drug overdose during the first year of the COVID-19 pandemic were identified. These findings may inform interventions that address macroenvironmental changes associated with the pandemic.

## Key Points

**Question:**

Were there changes in the rate and characteristics of deaths from drug overdose before vs during the COVID-19 pandemic in Rhode Island?

**Findings:**

In this population-based cohort study of 470 adults who died of drug overdose in Rhode Island from January 1 to August 31 in 2019 and 2020, rates of death from overdose among men, deaths involving synthetic opioids, and deaths occurring in personal residences increased significantly in 2020 compared with 2019. Deaths due to overdose also increased among people experiencing job loss and in subgroups with mental health diagnoses.

**Meaning:**

These findings suggest that policy and service delivery interventions that are responsive to emerging characteristics of deaths from drug overdose are needed to curtail these deaths.

## Teaser

This cohort study compares the rates and characteristics of deaths from drug overdose before vs during the COVID-19 pandemic in Rhode Island.

## Introduction

The syndemic of COVID-19 and deaths from drug overdose in the US continues to evolve. Although the numbers of COVID-19 cases and deaths have trended downward in the US since a peak in January 2021,^1^ provisional data indicate that the number of deaths from drug overdose has continued to increase.^2^ Macroenvironmental changes that began during the COVID-19 pandemic, such as increased physical isolation,^3,4^ mental health stressors,^5,6^ economic insecurity,^7,8^ and increased lethality of the drug supply,^2,9^ persist and may be associated with the continuing increase in overdose-related mortality nationwide. Although a few recent studies in the US^10,11,12,13^ have examined characteristics of deaths from overdose during the COVID-19 pandemic (primarily at the city level), scant data are currently available on the causes of the increase in these deaths during the COVID-19 pandemic or on the subpopulations at elevated risk.

Rhode Island has been particularly affected by the syndemic of COVID-19 and deaths from overdose. In December 2020, Rhode Island had the highest rate of COVID-19 cases and deaths per 100 000 population in the country,^14^ and preliminary data indicate that the rate of these deaths from overdose in the state in 2020 reached an all-time high. The Rhode Island Data Ecosystem, established in 2016, presents a unique opportunity to investigate characteristics of deaths from drug overdose by leveraging multiple statewide databases that are linked anonymously at the person level. The purpose of this study was to compare the characteristics of deaths from drug overdose during the first 8 months of 2020 with those during the same period in 2019 and to evaluate subpopulations at risk during 2020.

## Methods

### Study Design and Data Sources

We used data from the Rhode Island Data Ecosystem to conduct a population-based, retrospective cohort study of deaths from overdose in Rhode Island from January 1 to August 31, 2019, and from January 1 to August 31, 2020. The Data Ecosystem is an integrated analytics database that consists of anonymized, person-level data from multiple state agencies that are linked using a robust anonymization and person-matching process.^15,16^ In the present study, we used linked, statewide databases from 4 sources: the Rhode Island Department of Health, Medicaid claims and enrollment, the Department of Labor and Training, and the Homeless Management Information System. This study did not require oversight from an institutional review board according to 45 CFR §46 because it involved the analysis of preexisting, deidentified data from deceased individuals and did not involve living human participants. This study followed the Strengthening the Reporting of Observational Studies in Epidemiology (STROBE) reporting guideline for cohort studies.

### Key Variables

The primary outcome of interest in this study was the rate and characteristics of unintentional drug-related deaths due to overdose occurring in Rhode Island from January 1 to August 31, 2019, and from January 1 to August 31, 2020. Unintentional drug-related deaths due to overdose were identified by the Office of State Medical Examiners.^17,18^ Identified and confirmed unintentional drug-related deaths due to overdose were matched with data from the Center for Vital Records, which is managed by the Rhode Island Department of Health, and were transferred to the Data Ecosystem, as described above.

We obtained the sex, age, race and ethnicity, marital status, and veteran status of individuals who died of overdose from the Center for Vital Records. Drugs contributing to the cause of death (categorized as methadone hydrochloride, natural or semisynthetic opioids, synthetic opioids, heroin, cocaine, psychostimulants, and tobacco) and the location of death (categorized as hospital inpatient, hospital outpatient, personal residence, and other location) were extracted from the Office of State Medical Examiners cause of death and location of death fields, respectively, and were sourced through the Center for Vital Records.

Housing insecurity in the 12 months before death was ascertained from the Rhode Island Homeless Management Information System. This binary (yes or no) variable indicated any prior 12-month use of services reported to the information system from more than 40 in-state service providers. Job loss (yes or no) and earned wage data (categorized as <100% vs ≥100% of the federal poverty level) in the 12 months before death were ascertained from the Rhode Island Department of Labor and Training. Any record of receipt of income assistance payments in the 12 months before death (including unemployment insurance, pandemic unemployment assistance, and temporary disability insurance) was used as an indicator for job loss because loss of a job owing to circumstances beyond employee control is the primary qualifier in eligibility for income assistance.

In subgroup analyses, we examined behavioral health treatment and diagnosis claims among individuals who died of drug overdose who were enrolled in the state Medicaid program at any time during the 12 months before death. Using Medicaid claims and enrollment data, we examined the presence (yes or no) of the following behavioral health treatment and diagnosis claims in the 12 months before death: anxiety and fear-related disorders, depression, outpatient mental health treatment, inpatient mental health treatment, opioid use disorder, alcohol use disorder, any substance use disorder, and nonfatal overdose. We also examined the presence (yes or no) of any treatment with opioid agonist therapy (ie, methadone or buprenorphine hydrochloride) in the 3 months before death (eTable in the Supplement gives definitions and diagnosis codes).

### Statistical Analyses

We compared characteristics of individuals who died during the 2019 observational period with those of individuals who died during 2020 observation periods. Across characteristics, we calculated the incidence rate per 100 000 person-years and the absolute and percentage rate changes between observation periods. The total person-time units observed were derived from the 2019 American Community Survey (ACS) 1-Year Estimates Data Profiles from the US Census Bureau.^19^ Rates calculated for sex, age, race and ethnicity, marital status, and veteran status were adjusted to ACS-estimated population size; rates corresponding to the cause and location of death and socioeconomic factors used the ACS-estimated state total population. We compared the incident rate of deaths from overdose by these demographic and death-related characteristics between the 2019 and 2020 observation periods (Table 1). Counts with fewer than 5 deaths were suppressed in compliance with Rhode Island Department of Health data reporting policies.

**Table 1.** Count, Incidence Rate, and Rate Change of Unintentional Deaths From Drug Overdose Across Study Population Characteristics in Rhode Island From January to August in 2019 and 2020

Characteristic	Individuals, No. (%)		*P* value	Rate, per 100 000 PY^a^		Absolute rate change, per 100 000 PY	Change in rate, %	*P* value
	2019	2020		2019	2020			
All	206 (100)	264 (100)	NA	29.2	37.4	8.2	28.1	.009
Sex								
Male	149 (72)	204 (77)	.26	43.2	59.2	16.0	37	.003
Female	57 (28)	60 (23)	.26	15.8	16.6	0.8	5.1	.85
Age group, y								
18-19	<5^b^	<5^b^	>.99	NA^b^	NA^b^	NA^b^	NA^b^	NA^b^
20-29	30 (15)	33 (13)	.61	29.9	32.9	3.0	10	.80
30-39	57 (28)	70 (27)	.86	61.0	74.9	13.9	22.8	.29
40-49	49 (24)	61 (23)	.95	61.3	76.3	15.0	24.5	.29
50-59	50 (24)	71 (27)	.59	51.6	73.3	21.7	42.1	.07
60-69	18 (9)	25 (9)	.92	19.4	27.0	7.5	39.2	.36
≥70	<5^b^	<5^b^	.82	NA^b^	NA^b^	NA^b^	NA^b^	NA^b^
Race and ethnicity								
Hispanic	23 (11)	27 (10)	.86	20.0	23.5	3.5	17.5	.67
Non-Hispanic								
Black	25 (12)	23 (9)	.29	61.3	56.4	−4.9	−8.0	.89
White	155 (75)	210 (80)	.32	31.0	42.0	11.0	35.5	.005
Other or unknown^c^	<5^b^	<5^b^	>.99	NA^b^	NA^b^	NA^b^	NA^b^	NA^b^
Marital status								
Single	126 (61)	162 (61)	>.99	54.8	70.4	15.6	28.5	.04
Married	29 (14)	45 (17)	.46	11.3	17.6	6.3	55.8	.08
Divorced	43 (21)	51 (19)	.76	66.0	78.3	12.3	18.6	.47
Other status	8 (4)	6 (2)	.45	NA^b^	NA^b^	NA^b^	NA^b^	NA^b^
Veteran	13 (6)	18 (7)	.98	NA^b^	NA^b^	NA^b^	NA^b^	NA^b^
Contributing cause of death^d^								
Methadone	19 (9)	36 (14)	.18	NA^b^	5.1	NA^b^	NA^b^	NA^b^
Natural or semisynthetic opioids	26 (13)	36 (14)	.86	3.7	5.1	1.4	37.8	.25
Synthetic opioids^e^	147 (71)	200 (76)	.33	20.8	28.3	7.5	36.1	.005
Heroin	11 (5)	<5^b^	.02	NA^b^	NA^b^	NA^b^	NA^b^	NA^b^
Cocaine	99 (48)	129 (49)	.94	14.0	18.3	4.2	30.7	.06
Psychostimulants	9 (4)	21 (8)	.16	NA^b^	3.0	NA^b^	NA^b^	NA^b^
Benzodiazepines	22 (11)	32 (12)	.74	3.1	4.5	1.4	45.2	.22
Alcohol	59 (29)	70 (27)	.68	8.4	9.9	1.6	17.9	.38
Tobacco	17 (8)	37 (14)	.07	NA^b^	5.2	NA^b^	NA^b^	NA^b^
Location of death								
Hospital								
Inpatient	24 (12)	16 (6)	.047	3.4	NA^b^	NA^b^	NA^b^	NA^b^
Outpatient	67 (33)	70 (27)	.19	9.5	9.9	0.4	4.2	.86
Personal residence	93 (45)	139 (53)	.13	13.2	19.7	6.5	49.2	.003
Other	22 (11)	39 (15)	.24	3.0	4.7	1.7	56.7	.13
Socioeconomic factor^f^								
Housing insecurity	20 (10)	20 (8)	.51	2.8	2.8	0.0	0.0	>.99
Job loss	16 (8)	41 (16)	.01	NA^b^	6.5	NA^b^	NA^b^	NA^b^
FPL 100% or less^g^	25 (49)	38 (49)	>.99	3.5	5.4	1.8	54.3	.13

Abbreviations: FPL, federal poverty level; NA, not applicable; PY, person-years.

^a^ All rates and denominators were derived from the 2019 American Community Survey 1-Year Estimates Data Profiles.^19^

^b^ Cells with less than 5 deaths are suppressed in compliance with Rhode Island Department of Health data reporting policies. Rates based on less than 20 deaths are not considered reliable and are not reported.

^c^ Asian, Native American, mixed or other race, or unknown racial background.

^d^ Drugs contributing to the cause of death were not mutually exclusive.

^e^ Other than methadone.

^f^ Twelve months before death.

^g^ Wage data were available for 51 individuals who died of overdose in 2019 and 77 in 2020 from the Rhode Island Department of Health Center for Vital Records, Homeless Management Information System, and Rhode Island Department of Labor and Training.

In subgroup analyses, we compared behavioral health treatment and diagnosis claims among individuals who died of overdose in Rhode Island and were enrolled in Medicaid during the 12 months before death (Table 2). In additional exploratory analyses, we compared the frequency of select 2-factor combinations of behavioral health treatment and diagnosis claims between the 2019 and 2020 observation periods to assess potential changes in overdose risk within key subpopulations (Table 3). Two-factor combinations with cells containing fewer than 11 deaths (ie, 5 variable combinations) are not presented in compliance with Rhode Island Medicaid data reporting policies. All *P* values are 2 tailed and were computed using Fisher exact tests because this approach provides a conservative and reliable test of statistical significance when individual observations are independent and sample sizes are small.^20,21^ *P* ≤ .05 indicated statistical significance. All statistical analyses were performed using R, version 3.6.1 (R Program for Statistical Computing).

**Table 2.** Behavioral Health Treatment and Diagnosis Claims Among Individuals Who Died of Drug Overdose and Were Medicaid Beneficiaries in Rhode Island From January to August in 2019 and 2020

Diagnosis or treatment	Individuals, No. (%)^a^		*P* value
	2019 (n = 121)	2020 (n = 150)	
Psychiatric diagnoses^b^			
Anxiety and fear-related disorders	53 (44)	80 (53)	.15
Depression	55 (45)	80 (53)	.24
Mental health treatment			
Outpatient	61 (50)	70 (47)	.62
Inpatient	13 (11)	20 (13)	.65
Substance use–related diagnoses^b^			
Disorder			
Opioid use	59 (49)	76 (51)	.85
Alcohol use	55 (45)	60 (40)	.44
Any substance use	92 (76)	114 (76)	>.99
Nonfatal overdose	16 (13)	21 (14)	>.99
Treatment with opioid agonist therapy^c^			
Methadone	21 (17)	25 (17)	>.99
Buprenorphine	16 (13)	29 (19)	.24

^a^ Source is Rhode Island Data Ecosystem Medicaid data.

^b^ Indicates 12 months before death.

^c^ Indicates 3 months before death.

**Table 3.** Two-Factor Combinations of Characteristics and Behavioral Health Treatment and Diagnosis Claims Among Individuals Who Died of Drug Overdose and Were Medicaid Beneficiaries in Rhode Island by Change in Proportion From January to August in 2019 and 2020^a^

Factors 1 and 2	Individuals, No. (%)		Change, %	*P* value
	2019 (n = 121)	2020 (n = 150)		
Anxiety, aged 50-59 y	11 (9)	29 (19)	113	.03
Anxiety, aged 40-49 y	11 (9)	22 (15)	61	.23
Anxiety, male	28 (23)	55 (37)	58	.02
Anxiety, died as hospital outpatient	15 (12)	29 (19)	56	.17
Anxiety, single	31 (26)	54 (36)	41	.09
Anxiety, died at residence	25 (21)	33 (22)	6	.91
Depression, aged 50-59 y	12 (10)	26 (17)	75	.11
Depression, male	27 (22)	57 (38)	70	.008
Depression, died as hospital outpatient	14 (12)	28 (19)	61	.15
Depression, died at residence	26 (21)	35 (23)	9	.83
Depression, single	38 (31)	51 (34)	8	.75
Opioid use disorder, died as hospital outpatient	16 (13)	30 (20)	51	.19
Opioid use disorder, died at residence	31 (26)	31 (21)	−19	.41
Any substance use disorder, died as hospital outpatient	25 (21)	41 (27)	32	.26
Any substance use disorder, male	60 (50)	85 (57)	14	.30
Any substance use disorder, died at residence	46 (38)	51 (34)	−11	.58
Alcohol use disorder, died as hospital outpatient	16 (13)	25 (17)	26	.54
Alcohol use disorder, male	38 (31)	46 (31)	−2	>.99
Alcohol use disorder, died at residence	20 (17)	24 (16)	−3	>.99
Mental health treatment, died as hospital outpatient	19 (16)	25 (17)	6	.96
Mental health treatment, died at residence	24 (20)	30 (20)	1	>.99

^a^ Two-factor combinations with cells containing less than 11 deaths are not presented in compliance with Rhode Island Medicaid data reporting policies. Source is Rhode Island Data Ecosystem Medicaid data.

## Results

A total of 470 individuals who died of overdose were included in the analysis (353 men [75%] and 117 women [25%]; mean [SD] age, 43.5 [12.1] years). The rate of deaths from overdose in Rhode Island increased 28.1%, from 29.2 per 100 000 person-years during the observation period in 2019 to 37.4 per 100 000 person-years in 2020 (*P* = .009). In the 2020 observation period, those who died of overdose were primarily men (204 of 264 [77%]) and non-Hispanic White individuals (210 of 264 [80%]). The count, incidence rate per 100 000 person-years, and rate change of unintentional deaths due to drug overdose across characteristics, stratified by observation period, are presented in Table 1.

Although the proportions were unchanged for most variables compared with 2019, the rate of deaths from overdose during 2020 increased significantly among men (43.2 vs 59.2 per 100 000 person-years; *P* = .003), non-Hispanic White persons (31.0 vs 42.0 per 100 000 person-years; *P* = .005), and those who were single (54.8 vs 70.4 per 100 000 person-years; *P* = .04). The rate of deaths from overdose involving synthetic opioids increased significantly in 2020 compared with 2019 (20.8 vs 28.3 per 100 000 person-years; *P* = .005), as did the rate of deaths from overdose pronounced in a personal residence (13.2 vs 19.7 per 100 000 person-years; *P* = .003). In the 2020 observation period, the proportion of deaths from overdose pronounced in the hospital inpatient setting significantly decreased (24 of 206 [12%] vs 16 of 264 [6%]; *P* = .047), as did the proportion of deaths from overdose involving heroin (11 of 206 [5%] vs <5% [exact value suppressed]; *P* = .02). Conversely, there was a significant increase in the proportion of deaths from overdose among persons experiencing job loss (16 of 206 [8%] vs 41 of 264 [16%]; *P* = .01). The proportion of deaths from overdose occurring in personal residences also increased, but the difference was not significant (93 of 206 [45%] vs 139 of 264 [53%]; *P* = .13)

Among all individuals with death due to overdose in Rhode Island, 271 (58%) were Medicaid beneficiaries during the observation periods. Among Medicaid beneficiaries, we identified no significant change between the 2019 and 2020 periods in the proportion of deaths from overdose among individuals with claims for psychiatric diagnoses (eg, anxiety and fear-related disorders: 53 of 121 [44%] vs 80 of 150 [53%]; *P* = .15; depression: 53 of 121 [45%] vs 80 of 150 [53%]; *P* = .24), substance use–related diagnoses (eg, any substance use disorder: 92 of 121 [76%] vs 114 of 150 [76%]; *P* > .99), or treatment with opioid agonist therapy before death (eg, methadone: 21 of 121 [17%] vs 25 of 150 [17%]; *P* > .99). Behavioral health treatment and diagnosis claims for individuals who died of drug overdose and were Medicaid beneficiaries in Rhode Island, stratified by observation period, are presented in Table 2.

In exploratory analyses examining the frequency of 2-factor combinations of characteristics and behavioral health treatment and diagnosis claims among Medicaid beneficiaries in the 2019 and 2020 observation periods, we documented significant increases in the proportion of those aged 50 to 59 years with past 12-month anxiety diagnoses (11 of 121 [9%] vs 29 of 150 [19%]; *P* = .03), men with depression (27 of 121 [22%] vs 57 of 150 [38%]; *P* = .008), and men with anxiety (28 of 121 [23%] vs 55 of 150 [37%]; *P* = .02). Two-factor combinations of characteristics and behavioral health treatment and diagnosis claims among those who died due to drug overdose and were Medicaid beneficiaries in Rhode Island, stratified by observation period, are presented in Table 3.

## Discussion

During the first 8 months of 2020, the rate of deaths due to overdose increased 28% in Rhode Island compared with the same period in 2019. In this statewide analysis of multiple linked state administrative databases, we identified several changing characteristics of individuals who died from drug overdose in Rhode Island during 2020 vs 2019. To our knowledge, this study is among the first statewide analyses to evaluate evolving characteristics of deaths from overdose in 2020.

Our finding that the rate of deaths from overdose increased in 2020 is consistent with trends in national surveillance data that indicate that although rates of death from overdose were increasing through the end of 2019, rates of deaths due to overdose further increased during the beginning of the COVID-19 pandemic.^2,9^ Our findings are also consistent with preliminary findings from San Francisco (January 1 through April 18, 2020)^10^ and Indianapolis (suspected overdose events from January 1, 2019, through July 24, 2020)^11^; both studies reported substantial increases in deaths due to overdose during the initial months of the pandemic. Studies examining trends in overdose-related emergency medical services^22,23^ and emergency department visits^24^ also documented similar surges in 2020 observation periods. Of note, however, our findings are divergent from preliminary research from Philadelphia^12^ that did not identify changes in unintentional deaths from opioid-related overdose when comparing April to June 2019 with April to June 2020. These divergent findings in overdose fatality trends at the city level underscore that although deaths from overdose increased nationwide during early 2020, sources of heterogeneity at the region and state levels warrant further investigation.

Several researchers^25,26,27,28,29^ have hypothesized that the social distancing and stay-at-home orders that were necessary to limit the spread of COVID-19 would be associated with an increased risk of overdose owing to exacerbated social isolation and despair, diminished social support, and an increase in use of drugs while alone. Our finding that most individuals who died from overdose in 2020 died in their personal residence (53%) supports this hypothesis. We also found significant decreases in the proportion of deaths pronounced in the hospital inpatient setting (ie, individuals who were admitted alive but subsequently died), which is consistent with national trends indicating decreases in hospital admissions not related to COVID-19 in 2020.^30^ These findings may also be associated with increased reluctance to use emergency medical services and emergency departments during the pandemic,^31^ persisting hesitancy in calling 911 owing to fear of arrest or homicide charges,^32,33^ or increased drug supply lethality.

Given the observed increase in deaths from overdose occurring in personal residences, we recommend that states bolster overdose reversals in these settings by strengthening Good Samaritan law protections for those who call 911.^32,34^ We also recommend that states establish pilot overdose prevention sites to provide a safer, supervised environment for drug use to prevent deaths from overdose catalyzed by isolation.^35,36,37,38^ In addition, treatment programs and recovery centers should safely prioritize in-person recovery services^39^ to enhance social supports for individuals experiencing social isolation and elevated overdose risk.

Our finding that 58% of individuals who died of overdose and were Medicaid beneficiaries in Rhode Island in 2020 had prior diagnoses of anxiety and fear-related disorders (53%) and depression (53%) is consistent with prior literature documenting a high prevalence of comorbidly diagnosed anxiety and depression among individuals who died of overdose.^40,41^ We also found that the proportion of deaths from overdose increased within specific subgroups of individuals with psychiatric diagnoses (ie, persons aged 50-59 years with anxiety, men with depression, and men with anxiety). These findings may suggest an increase in deaths from overdose associated with increased social isolation and other macroenvironmental features of the pandemic; however, these factors were not analyzed in the present study. These results also suggest that persons with mental health conditions are at elevated risk of a fatal overdose^40,41^ and may have had diminished access to protective resources, such as behavioral health care, social support, and harm reduction supplies, during the COVID-19 pandemic.^42,43^ Given the increase in deaths from overdose in subgroups of individuals with psychiatric diagnoses found in this study, we recommend that primary care offices, community mental health organizations, outpatient behavioral health, and inpatient psychiatric hospitals establish on-demand buprenorphine induction to increase treatment access among individuals with mental health conditions^16,44,45,46^ and that states include and fund trauma-informed mental health services in treatment programs for alcohol and substance use disorders.^47,48,49,50^ Of importance, the efficacy and impact of state responses to the overdose crisis are contingent, at least in part, on the operational efficiency and capacity of recovery support services and behavioral health care systems, which encountered challenges during the COVID-19 pandemic.^51,52,53^ By also enhancing the investment in these essential programs, preparedness and response time during the next pandemic or natural disaster may be improved and service access and continuity of care for those at greatest risk of deaths from overdose may increase.

We found that the proportion of deaths from overdose among individuals who recently experienced job loss (as indicated by receipt of income assistance payments) increased significantly. This finding may be associated with the more than 4-fold increase in unemployment in the general population during the pandemic.^54^ Although some prior research suggests an association between synchronous income assistance payments and subsequent overdose through the “check effect,”^55,56,57,58^ research investigating this phenomenon at the neighborhood level in Rhode Island did not identify an association between the proportion of residents receiving monthly income assistance and excess mortality at the beginning of the month, although excess mortality was associated with the proportion of residents living in unaffordable housing.^59^ Correspondingly, strategies that alleviate structural stressors that co-occur with income assistance payments (ie, rent or mortgage payments) by expanding affordable housing availability may be associated with a reduction in deaths from overdose at the beginning of the month.^60^ States should also enhance workforce development and training initiatives for persons with substance use disorders and those in recovery.^61,62^

Our study showed significant increases in the rate of deaths from overdose involving synthetic opioids, such as fentanyl. This evidence is consistent with national surveillance data,^9^ which indicate that synthetic opioids (primarily illicitly manufactured fentanyl) appear to be the primary factor associated with the increased rate of deaths from overdose death from 2019 to 2020. We also documented a significant decrease in the proportion of deaths from overdose involving heroin. This finding is consistent with a study^63^ reporting reductions in heroin-involved deaths from overdose from 2018 to 2020 in Massachusetts. Given the observed increase in the rate of deaths from overdose involving synthetic opioids such as fentanyl and the corresponding increased toxicity of drug supplies, we recommend that states respond to this environment of increased risk by embracing proven strategies to reduce deaths from overdose. Specifically, states should establish sustainable funding to expand distribution of naloxone hydrochloride^64,65^ and fentanyl test strips,^66,67,68,69,70^ ensuring that resources are allocated to subgroups at elevated risk; prioritize and fund medication-first treatment approaches (eg, low-threshold buprenorphine treatment programs,^46,71^ audio-only telehealth for initiation of buprenorphine treatment^72,73^) that reduce barriers to enrollment and continued engagement in treatment; and develop harm reduction messaging campaigns targeting those affected by isolation, mental health conditions, and economic insecurity.

The key findings identified in the present study correspond with 4 primary environmental changes associated with the COVID-19 pandemic: increased isolation,^3,4^ mental health stressors,^5,6^ economic insecurity,^7,8^ and lethality of the drug supply.^2,9^ The Figure shows key findings and recommendations corresponding to environmental changes that occurred during the COVID-19 pandemic. We demarcate our recommendations corresponding to needed changes in service delivery (ie, organizational policies and procedures) and in statewide policy to reduce deaths from overdose given these emerging trends.

**Figure.**
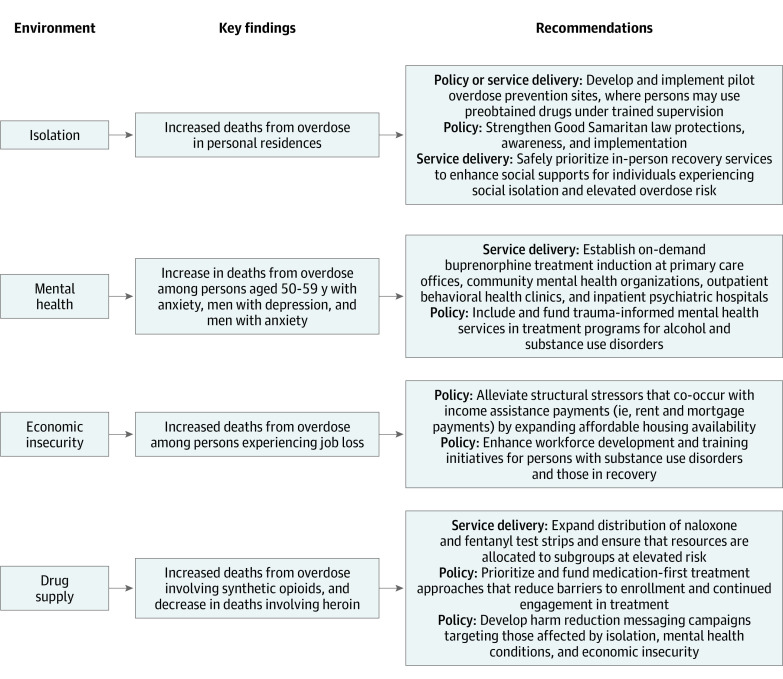
Summary of Key Findings and Recommendations Corresponding to Macroenvironmental Changes That Occurred During the COVID-19 Pandemic

### Limitations

This study has limitations. First, because of the small sample sizes, the risk for types I and II error was increased. Second, individual characteristics or behavioral health treatment and diagnosis claims may have been misclassified through either incomplete data or linkage error. Although the person-matching systems and data environment leveraged for the present study were robust, error owing to misclassification is possible and would bias results toward the null. Third, our findings may have limited generalizability outside Rhode Island, which has higher overdose mortality and higher rates of Medicaid enrollment; these factors may be associated with the characteristics for the individuals who died of overdose. Fourth, the observation period was limited to 16 total months because data through August 2020 were the most recently available data at the time of analysis; it is possible that some differences identified between the 2019 and 2020 observation periods reflect typical year-to-year variations that cannot be characterized owing to our limited time frame. Fifth, the observed reduction in deaths pronounced in the hospital inpatient setting and the observed increase in deaths among persons experiencing job loss may reflect underlying trends in the general population during the COVID-19 pandemic.^30,54^ Sixth, our counts of overdose deaths among individuals experiencing job loss, housing insecurity, and behavioral health needs may be underestimated in the 2020 observation period owing to increased service demand and diminished service accessibility during the initial months of the COVID-19 pandemic.

## Conclusions

In this cohort study of deaths from overdose occurring during the first 8 months of 2019 and 2020 in Rhode Island, the rate of deaths from overdose increased in 2020 compared with the same period in 2019, and we identified several evolving characteristics of deaths from drug overdose. These characteristics appear to correspond with environmental changes that occurred during the COVID-19 pandemic, including increased isolation, mental health stressors, economic insecurity, and drug supply lethality. These findings suggest that targeted opportunities exist to adapt service delivery and state policies in response to the increase in the rate of deaths from overdose.

## Notes

Supplement. **eTable.** Variable Code Details for Behavioral Health Treatment and Diagnosis Claims Sourced From Rhode Island Medicaid Claims and Enrollment Data
