# Supplementary material for: Comparison of Characteristics of Deaths From Drug Overdose Before vs During the COVID-19 Pandemic in Rhode Island
Source: JAMA Netw Open. 2021 Sep 17;4(9):e2125538. doi: 10.1001/jamanetworkopen.2021.25538 (PMC8449276; doi:10.1001/jamanetworkopen.2021.25538)
Supplement: Supplement. — eTable. Variable Code Details for Behavioral Health Treatment and Diagnosis Claims Sourced From Rhode Island Medicaid Claims and Enrollment Data [file jamanetwopen-e2125538-s001.pdf]

Supplementary Online Content

Macmadu A, Batthala S, Correia Gabel AM, et al. Comparison of characteristics of deaths from drug overdose before vs during the COVID-19 pandemic in Rhode Island. *JAMA Netw Open*. 2021;4(9):e2125538. doi:10.1001/jamanetworkopen.2021.25538

eTable. Variable Code Details for Behavioral Health Treatment and Diagnosis Claims Sourced From Rhode Island Medicaid Claims and Enrollment Data

This supplementary material has been provided by the authors to give readers additional information about their work.

©2021 Macmadu A et al. *JAMA Network Open*.

eTable. Variable Code Details for Behavioral Health Treatment and Diagnosis Claims Sourced From Rhode Island Medicaid Claims and Enrollment Data

Variable	Code details
Anxiety and fear related disorders	F930, F940, F4000, F4001, F4002, F4010, F4011, F40210, F40218, F40220, F40228, F40230, F40231, F40232, F40233, F40240, F40241, F40242, F40243, F40248, F40290, F40291, F40298, F408, F409, F410, F411, F413, F418, F419, F064
Depression	F320, F321, F322, F323, F324, F328, F3281, F3289, F329, F330, F331, F332, F333, F3341, F338, F339, F341, F0631, F0632
Mental health treatment (outpatient)	[Procedure_Code] IN ('H0046', '90834', '90847', '97150', '90791', '90837', '90833', '90834', '90836', '90853', '90785', '90792', '99408', '99409', 'H0049') AND [Diagnosis_1] NOT IN ('F11181', '30472', '30471', '30551', '30470', '30553', '30400', '30550', '30473', '30402', '30403', '30552', '30401', 'F1119', 'F11288', 'F1194', 'F11259', 'F11251', 'F11120', 'F11129', 'F11988', 'F11122', 'F11920', 'F11151', 'F11182', 'F11222', 'F1111', 'F11250', 'F11951', 'F1199', 'F11229', 'F11950', 'F1114', 'F11150', 'F1193', 'F1124', 'F11188', 'F1129', 'F11929', 'F1110', 'F11281', 'F11959', 'F1121', 'F11221', 'F11921', 'F11982', 'F11220', 'F1123', 'F11282', 'F11121', 'F11159', 'F1120', 'F1190', 'F11922', 'F11981', 'F1111')
Mental health treatment (inpatient)[a]	Diagnosis_1 in the list of codes AND [IP_Flag] = '1' AND [Billing_Provider_NPI] NOT IN ('1992884787', '1841323094', '1093848327', '1164822474', '1801966510', '1851303614', '1821078932', '1821218587', '1134281280', '1003045782', '1013332014', '1093713174', '1104847946', '1124198676', '1164822474', '1245239680', '1265595433', '1316917222', '1356343651', '1366501603', '1386671535', '1437191061', '1487916136', '1609925122', '1659379410', '1659517787', '1700913944', '1952699985', '1124170444', '1144606807', '1205278223', '1205968419', '1235207044', '1265752737', '1326267063', '1346519733', '1356338248', '1558557629', '1629123021', '1659591261', '1679919203', '1871515783', '1881684736', '1912038738', '1982914909', '1992128540'
Opioid use disorder	F11181, 30472, 30471, 30551, 30470, 30553, 30400, 30550, 30473, 30402, 30403, 30552, 30401, F1119, F11288, F1194, F11259, F11251, F11120, F11129, F11988, F11122, F11920, F11151, F11182, F11222, F1111, F11250, F11951, F1199, F11229, F11950, F1114, F11150, F1193, F1124, F11188, F1129, F11929, F1110, F11281, F11959, F1121, F11221, F11921, F11982, F11220, F1123, F11282, F11121, F11159, F1120, F1190, F11922, F11981
Alcohol use disorder	G312, F10920, F10921, F10929, F1094, F10950, F10951, F10959, F1096, F1097, F10980, F10981, F10982, F10988, F1099, F1010, F10120, F10121, F10129, F1014, F10150, F10151, F10159, F10180, F10181, F10182, F10188, F1019, F1020, F10220, F10221, F10229, F10230, F10231, F10232, F10239, F1024, F10250, F10251, F10259, F1026, F1027, F10280, F10281, F10282, F10288, F1029
Any substance use disorder	F18150, F18151, F18159, F1817, F18180, F18188, F1819, F1820, F1821, F18220, F18221, F18229, F1824, F1897, F18980, F18988, F1899, F1910, F1911, F19120, F19121, F19122, F19129, F1914, F19150, F19151, F19159, F1916, F1917, F19180, F19181, F19182, F19188, F1919, F1920, F1921, F19220, F19221, F19222, F19229, F19230, F19231, F19232, F19239, F1924, F19250, F19251, F19259, F1926, F1927, F19280, F19281, F19282, F19288, F1929, F1990, F19920, F19921, F19922, F19929, F19930, F19931, F19932, F19939, F1994, F19950, F19951, F19959, F1996, F1997, F19980, F19981, F19982, F19988, F1999, F15121, F15122, F15129, F1514, F1529, F1590, F15920, F15921, F15922, F15929, F1593, F1594, F15950, F15951, F15959, F15980, F15981, F15982, F15988, F1599, F1610, F1611, F16120, F16121, F16122, F16129, F1614, F16150, F16151, F16159, F16180, F16183, F16188, F1619, F1620, F1621, F16220,

©2021 Macmadu A et al. *JAMA Network Open*.

	F16221, F16229, F1624, F16250, F16251, F16259, F16280, F16283, F16288, F1629, F1690, F16920, F16921, F16929, F1694, F16950, F16951, F16959, F16980, F16983, F16988, F1699, F17200, F17201, F17203, F17208, F17209, F17210, F17211, F17213, F17218, F17219, F17220, F17221, F17223, F17228, F17229, F17290, F17291, F17293, F17298, F17299, F1810, F1811, F18120, F18121, F18129, F1814, F18250, F18251, F18259, F1827, F18280, F18288, F1829, F1890, F18920, F18921, F18929, F1894, F18950, F18951, F18959, F13231, F13232, F13239, F1324, F13250, F13251, F13259, F1326, F1327, F13280, F13281, F13282, F13288, F1329, F1390, F13920, F13921, F13929, F13930, F13931, F13932, F13959, F1396, F1397, F13980, F13981, F13982, F13988, F1399, F1410, F1411, F14120, F14121, F14122, F14129, F1414, F14150, F14151, F14159, F14180, F14181, F14182, F14188, F1419, F1420, F1421, F14220, F14221, F14222, F14229, F1423, F1424, F14250, F14251, F14259, F14280, F14281, F14282, F14288, F1429, F1490, F14920, F14921, F14922, F14929, F1494, F14950, F14951, F14959, F14980, F14981, F14982, F14988, F1499, F1510, F1511, F15120, F15150, F15151, F15159, F15180, F15181, F15182, F15188, F1519, F1520, F1521, F15220, F15221, F15222, F15229, F1523, F1524, F15250, F15251, F15259, F15280, F15281, F15282, F15288, F10920, F10921, F10929, F1094, F10950, F10951, F10959, F1096, F1097, F10980, F10981, F10982, F10988, F1099, F1121, F1210, F1211, F12120, F12121, F12122, F12129, F12150, F12151, F12159, F12180, F12188, F1219, F1220, F1221, F12220, F12221, F12222, F12229, F1223, F12250, F12251, F12259, F12280, F12288, F1229, F1290, F12920, F12921, F12922, F12929, F1293, F12950, F12951, F12959, F12980, F12988, F1299, F1310, F1311, F13120, F13121, F13129, F1314, F13150, F13151, F13159, F13180, F13181, F13182, F13188, F1319, F1320, F1321, F13220, F13221, F13229, F13230, F13939, F1394, F13950, F13951, F1010, F1011, F10120, F10121, F10129, F1014, F10150, F10151, F10159, F10180, F10181, F10182, F10188, F1019, F1020, F1021, F10220, F10221, F10229, F10230, F10231, F10232, F10239, F1024, F10250, F10251, F10259, F1026, F1027, F10280, F10281, F10282, F10288, F1029, F1111
Nonfatal overdose	([ED_Flag] = '1' OR [Revenue_Code] IN ('0450', '0451', '0452', '0453', '0454', '0455', '0456', '0457', '0458', '0459')) AND [Diagnosis_Code] IN -- ICD-9 Codes ('96500', '96501', '96502', '96509', '9701', 'E8500', 'E8501', 'E8502', 'E9350', 'E9351', 'E9352', 'E9401', -- ICD-10 Codes 'T400X1A', 'T400X1D', 'T400X2A', 'T400X2D', 'T400X3A', 'T400X3D', 'T400X4A', 'T400X4D', 'T401X1A', 'T401X1D', 'T401X2A', 'T401X2D', 'T401X3A', 'T401X3D', 'T401X4A', 'T401X4D', 'T402X1A', 'T402X1D', 'T402X2A', 'T402X2D', 'T402X3A', 'T402X3D', 'T402X4A', 'T402X4D', 'T403X1A', 'T403X1D', 'T403X2A', 'T403X2D', 'T403X3A', 'T403X3D', 'T403X4A', 'T403X4D', 'T404X1A', 'T404X1D', 'T404X2A', 'T404X2D', 'T404X3A', 'T404X3D', 'T404X4A', 'T404X4D', 'T40601A', 'T40601D', 'T40602A', 'T40602D', 'T40603A', 'T40603D', 'T40604A', 'T40604D', 'T40691A', 'T40691D', 'T40692A', 'T40692D', 'T40693A', 'T40693D', 'T40694A', 'T40694D', 'T400X5A', 'T400X5D', 'T402X5A', 'T402X5D', 'T403X5A', 'T403X5D', 'T404X5A', 'T404X5D', 'T40605A', 'T40605D', 'T40695A', 'T40695D')
Methadone	ICD code: H0020
Buprenorphine	RED.[Theraputic_Class_Code] = '2808120005' AND NOT (RED.Product_Name IN ('BUTRANS', 'BELBUCA', 'BUPRENEX') OR RED.National_Drug_Code IN ('00409201232', '42023017905', '00093360040', '00093360140', '00093360240', '00093360340', '00093365640', '00093365740', '00093365840', '00093365940', '42858035340', '42858049340', '42858058640', '42858075040', '42858083940'))

[a]There are over 1500 Diagnosis_1 codes included in this definition. The authors will make these codes available upon request.

©2021 Macmadu A et al. *JAMA Network Open*.
